# Increased Ca_V_1.2 late current by a *CACNA1C* p.R412M variant causes an atypical Timothy syndrome without syndactyly

**DOI:** 10.1038/s41598-022-23512-2

**Published:** 2022-11-08

**Authors:** Junichi Ozawa, Seiko Ohno, Dario Melgari, Qi Wang, Megumi Fukuyama, Futoshi Toyoda, Takeru Makiyama, Masao Yoshinaga, Hiroshi Suzuki, Akihiko Saitoh, Tomohiko Ai, Minoru Horie

**Affiliations:** 1 Department of Cardiovascular Medicine, Shiga University of Medical Science, Seta-Tsukinowa, Otsu, Shiga 520-2192 Japan; 2 Department of Pediatrics, Niigata University Graduate School of Medical and Dental Sciences, Niigata, Japan; 3 Department of Bioscience and Genetics, National Cerebral and Cardiovascular Center, Suita, Japan; 4 Institute of Molecular and Translational Cardiology (IMTC), IRCCS Policlinico San Donato, 20097 San Donato Milanese, Milan, Italy; 5 Department of Physiology, China Medical University, Shenyang, Liaoning China; 6 Department of Physiology, Shiga University of Medical Science, Otsu, Japan; 7 Department of Cardiovascular Medicine, Kyoto University Graduate School of Medicine, Kyoto, Japan; 8 Department of Pediatrics, National Hospital Organization Kagoshima Medical Center, Kagoshima, Japan; 9 Department of Clinical Laboratory Medicine, Juntendo University School of Medicine, Tokyo, Japan; 10 Department of Medicine, Krannert Institute of Cardiology, Indiana University School of Medicine, Indianapolis, USA

**Keywords:** Genetics, Cardiology

## Abstract

Timothy syndrome (TS) is a rare pleiotropic disorder associated with long QT syndrome, syndactyly, dysmorphic features, and neurological symptoms. Several variants in exon 8 or 8a of *CACNA1C*, a gene encoding the α-subunit of voltage-gated Ca^2+^ channels (Ca_v_1.2), are known to cause classical TS. We identified a p.R412M (exon 9) variant in an atypical TS case. The aim of this study was to examine the functional effects of *CACNA1C* p.R412M on Ca_V_1.2 in comparison with those of p.G406R. The index patient was a 2-month-old female infant who suffered from a cardio-pulmonary arrest in association with prolonged QT intervals. She showed dysmorphic facial features and developmental delay, but not syndactyly. Interestingly, she also presented recurrent seizures from 4 months. Genetic tests identified a novel heterozygous *CACNA1C *variant, p.R412M. Using heterologous expression system with HEK-293 cells, analyses with whole-cell patch-clamp technique revealed that p.R412M caused late Ca^2+^ currents by significantly delaying Ca_V_1.2 channel inactivation, consistent with the underlying mechanisms of classical TS. A novel *CACNA1C* variant, p.R412M, was found to be associated with atypical TS through the same mechanism as p.G406R, the variant responsible for classical TS.

## Introduction

Timothy syndrome (TS) is a rare pleiotropic disorder associated with long QT syndrome (LQTS, type 8), congenital heart disease, syndactyly, dysmorphic features, immunodeficiency, intermittent hypoglycemia, and neurologic symptoms including autism, seizures, and intellectual disability^1,2^. TS is caused by missense variants in *CACNA1C*, the gene encoding the α-subunit of voltage-gated Ca^2+^ channels (Ca_V_1.2)^2,3^. A functional study showed that p.G406R in exon 8a, which is responsible for TS1, significantly slowed the voltage-dependent inactivation (VDI) kinetics, resulting in sustained late Ca^2+^ currents^1^. Although syndactyly is a common feature of the classical form of TS1, two atypical patients who showed severe cardiac deficits did not have syndactyly; furthermore they differed genetically, and thus were later categorized as TS2^4^. TS2 patients were found to carry heterozygous missense variants, p.G406R and p.G402S, in a mutually exclusive exon 8. Exon 8 is more predominantly expressed in the heart compared to exon 8a. It is thought that the different expression levels of two transcripts containing either exon 8a or 8 account for those different phenotypes; TS1 patients exhibit a more severe form of extra-cardiac features than TS2^1,4^.

We experienced a female infant who suffered from cardiac arrest due to Torsade de Pointes (TdP) in association with Timothy syndrome without syndactyly, which mimics TS2. The patient also presented developmental delay and was complicated with recurring seizure attacks. We identified a heterozygous de novo *CACNA1C* variant p.R412M that was located six amino acids downstream to G406R and between domain I-S6 (IS6) and α1 interacting domain (AID). The AID is known as the binding site for ancillary β-subunit, a potent modulator of voltage-dependent calcium channels. In the present study, we described the clinical phenotypes of the patient and analyzed the functional effects of p.R412M variant on Ca_V_1.2. We also conducted the functional assay of the p.G406R, a variant found in TS1.

## Results

### Clinical features of index patient

The patient was a 2-month-old female infant born after 37 weeks of gestation with a birth weight of 2730 g. Fetal bradycardia had been identified at gestational age of 33 weeks, but no cardiac rhythm disorder was noted at birth. Her face was characterized by dysmorphic features such as high arched palate, full cheeks, and congenital clasped thumb, but no syndactyly. At 2 months, she experienced her first episode of syncope due to repetitive TdPs that degenerated into VF.

**Figure 1:**
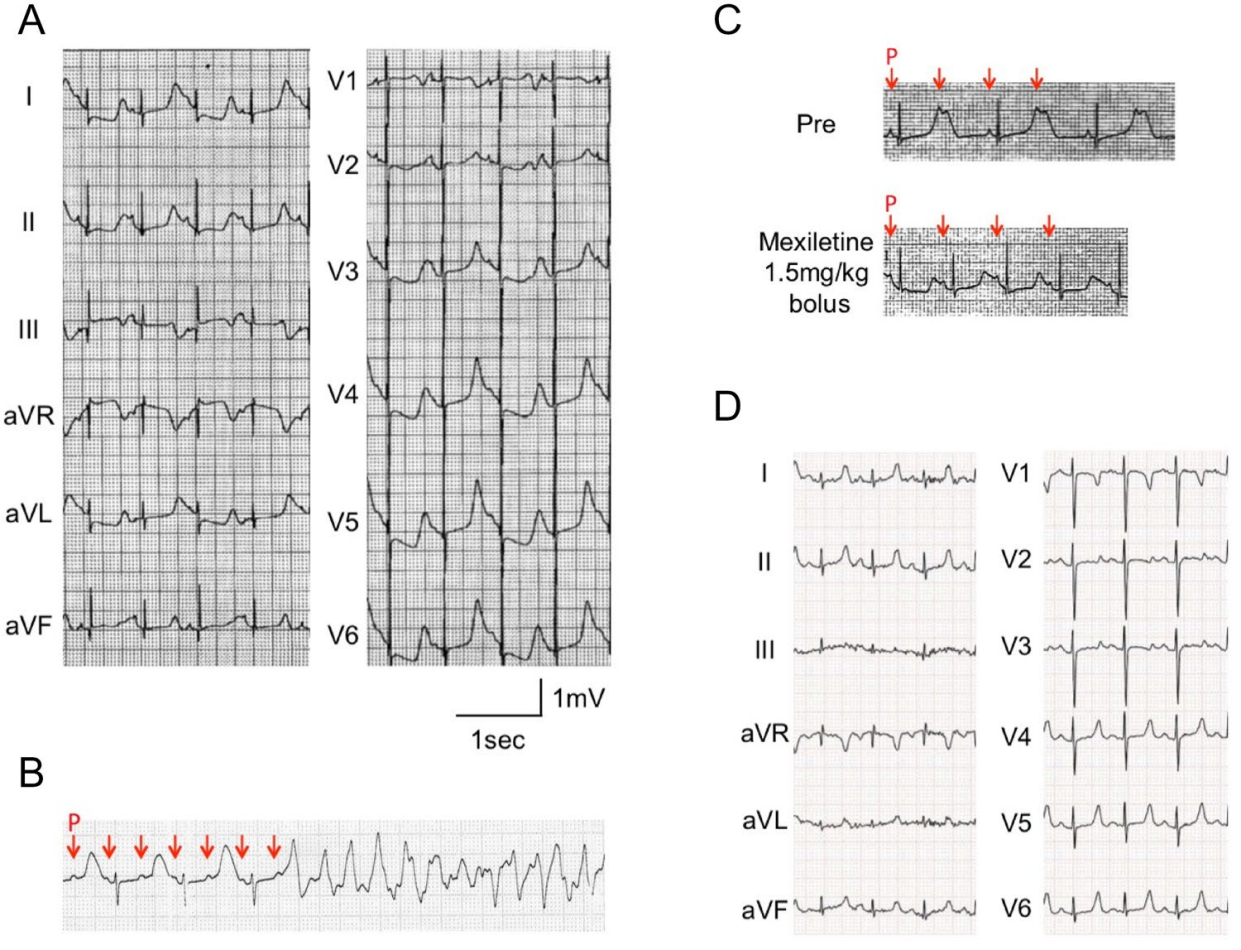
Electrocardiograms (ECG) of a 2-month-old female infant with Timothy syndrome, showing (**A**) T-wave alternans, (**B**) 2:1 atrio-ventricular block and torsades de pointes, (**C**) before and after the intravenous mexiletine, and (**D**) after oral administration of mexiletine and propranolol. Red arrows in (**B**) and (**C**) indicate P waves.

Her ECG revealed typical T wave alternans, markedly prolonged QT-intervals (RR = 570 ms, QT = 501 ms, QTc = 664 ms, Fig. 1A), 2:1 atrio-ventricular (AV) block, and recurrence of TdP (Fig. 1B). The echocardiography showed no congenital heart defects nor hypertrophy. We suspected LQTS type 3 (LQT3) from the T-wave morphology, depicting a late-onset peaked T-wave. Therefore, mexiletine was administered as an initial therapy. An intravenous injection of mexiletine (1.5 mg/kg) followed by the maintenance dose of oral mexiletine (30 mg/kg/day) resolved the 2:1 AV block to a 1:1 conduction (Fig. 1C). In addition, we started propranolol (2 mg/kg/day), which suppressed recurrence of TdP.

The patient also suffered from recurrent seizures unrelated to TdPs from 4 months after birth. An electroencephalogram at the age of 7 months displayed hypsarrhythmia. She showed severe developmental disability and hypotonia, and thus she was barely able to roll over at the age of 3. The patient was also diagnosed with autism spectrum disorder at the age of 2. There were no findings that indicated hypoglycemia or immunodeficiency related to TS.

With the medications, the patient’s ECG at the age of 5 showed slightly prolonged QTc (RR = 559 ms, QT = 346 ms, QTc = 462 ms; Fig. 1D). Since the pharmacotherapy successfully suppressed her TdP, implantable cardioverter-defibrillator (ICD) was not implanted. Unfortunately, the patient suddenly passed away at 5 years old during a nap. Her family history was negative for SCD, LQTS, arrhythmia, or neurological abnormalities.

### Genetic analysis

**Figure 2:**
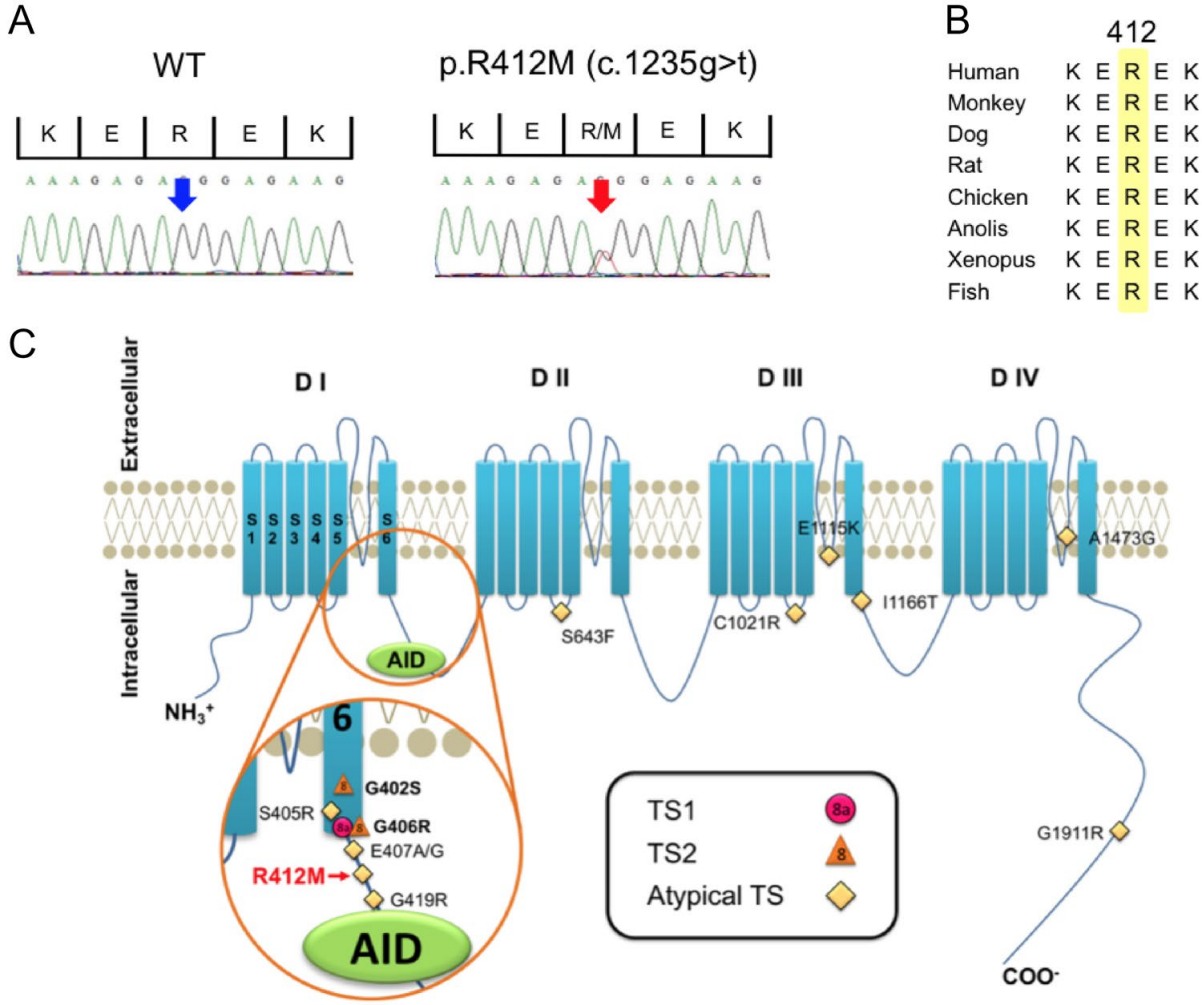
(**A**) Electropherograms of WT and p.R412M. (**B**) Alignment of p.R412M. (**C**) Topology of Ca_V_1.2 α-subunit (S, segment; D, domain) with classical TS (TS1, pink circles), TS2 (orange triangles), or atypical TS (yellow diamond) -related mutations. TS1. Timothy syndrome type 1; TS2, Timothy syndrome type 2; Atypical TS, Atypical Timothy syndrome; AID, α1 interacting domain.

Genetic tests using a gene panel as described in the “Methods” section identified a novel heterozygous missense variant p.R412M in *CACNA1C*. This variant was confirmed by the Sanger method (Fig. 2A). The arginine at position 412 is highly conserved among different species (Fig. 2B). The patient's parents were both negative for this variant, indicating a de novo mutation within this family. Their paternity and maternity were confirmed by screening 12 rare single nucleotide polymorphisms (data not shown).

Figure 2C illustrates a topology of *CACNA1C* in which known TS-related mutations identified to date are highlighted. The variant identified in this study is indicated in red (with an arrow). Arginine 412 is located in the inner loop of the membrane, between IS6 and binding site for ancillary β-subunit (AID). The p.R412M variant has not been previously reported in at least two online databases: TOGOVAR (https://togovar.biosciencedbc.jp/) and gnomAD (https://gnomad.broadinstitute.org/). The variants with functional evidence were confirmed as pathogenic based on the American College of Medical Genetics and Genomics (ACMG) guideline for the interpretation of sequence variants^5^.

### Functional analysis

#### Electrophysiological parameters

**Figure 3:**
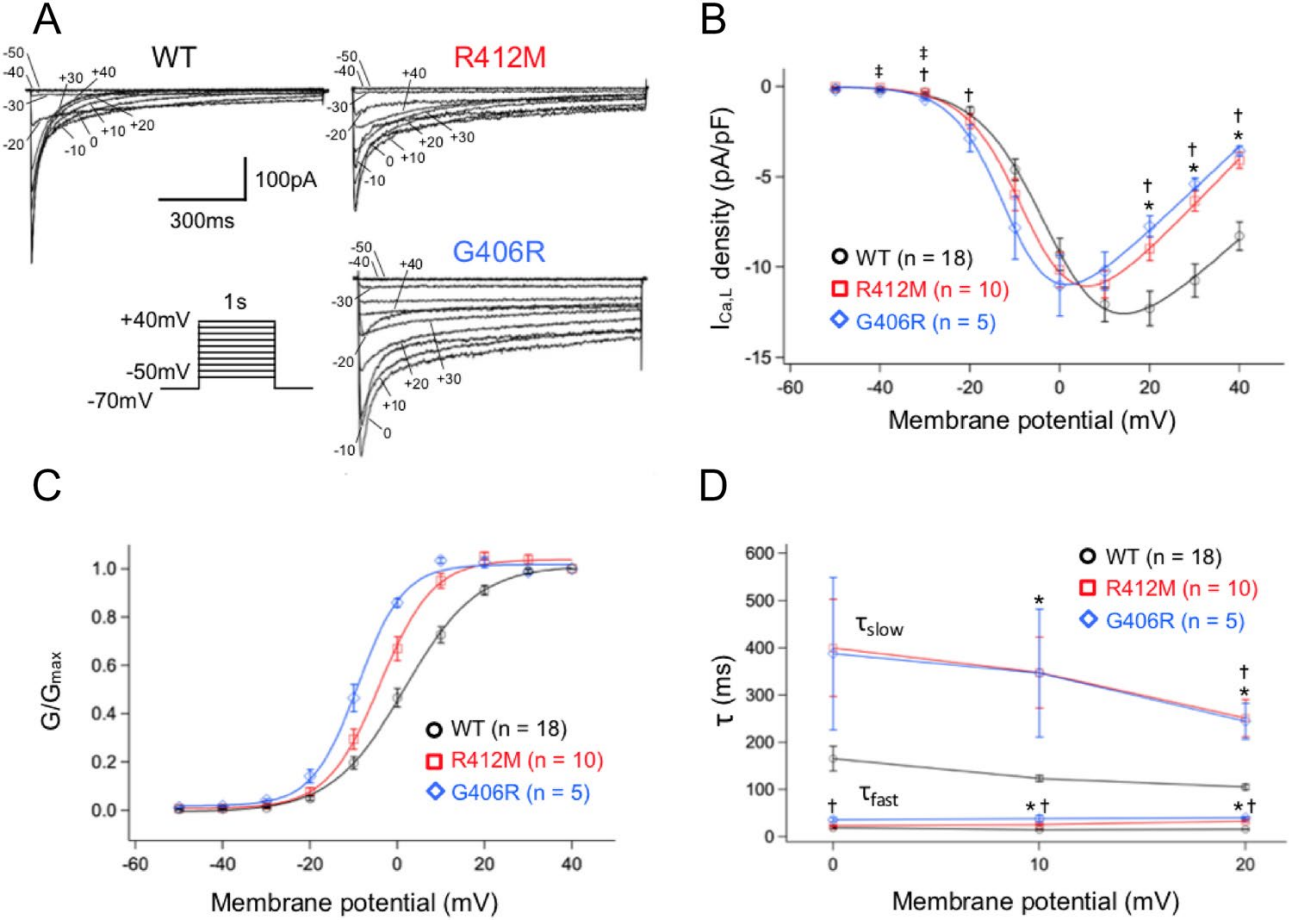
(**A**) Three sets of representative current traces for WT, R412M, and G406R I_Ca_ elicited by the protocol shown in the inset. (**B**) peak current density–voltage relationships, (**C**) steady state activation curves and (**D**) inactivation time constants for three different I_Ca_: WT (black circles, n = 18), R412M (red squares, n = 10) and G406R (blue diamonds, n = 5). *P < 0.05 R412M vs. WT. ^†^*P* < 0.05 G406R vs. WT. ^‡^*P* < 0.05 R412M vs. G406R.

We examined the electrophysiological characteristics of WT, R412M, and G406R Ca_V_1.2 channels. Figure 3A shows representative current traces recorded from HEK-293 cells transiently transfected with WT (left), R412M (right upper), or G406R (right lower) *CACNA1C*. Maximal peak current densities were not significantly different among the three types of cells (I_Ca,WT_: − 12.3 ± 0.95 pA/pF at + 20 mV I_Ca,R412M_: − 10.9 ± 0.75 pA/pF at + 10 mV, I_Ca,G406R_: 11.0 ± 1.7 pA/pF at + 10 mV; *p* = 0.42). In contrast, the inactivation decay of reconstituted Ca^2+^ currents was significantly slower in R412M- and G406R-transfected cells compared to those with WT (I_Ca,WT_). Table 1 summarizes the biophysical parameters measured from multiple cells.

**Table 1:** Biophysical parameters of Ca_v_1.2 WT, R412M and G406R channels.

Biophysical parameter		WT	R412M	G406R
Activation parameters		(n = 18)	(n = 10)	(n = 5)
Peak density (pA/pF)		− 12.3 ± 0.95	− 10.9 ± 0.75	− 11.0 ± 1.7
V_*h*_ (mV)		1.9 ± 1.3	− 3.7 ± 1.3*	− 9.2 ± 1.2^†^
* k*		7.8 ± 0.23	5.7 ± 0.23*	5.1 ± 0.40^†^
Conductance parameters		(n = 18)	(n = 10)	(n = 5)
V_rev_ (mV)		72.5 ± 1.3	62.8 ± 2.1*	60.8 ± 2.0^†^
Peak density (pS)		0.26 ± 0.025	0.22 ± 0.056	0.17 ± 0.013
Inactivation parameters		(n = 15)	(n = 12)	(n = 6)
I_Ca_ V_*h*_ (mV)		− 11.8 ± 1.4	− 4.1 ± 1.4*	− 10.0 ± 2.0
I_Ca_ *k*		− 8.2 ± 1.1	− 7.8 ± 0.65	− 6.6 ± 0.52^†^
I_Ba_ Vh (mV)		− 21.7 ± 6.3	− 12.1 ± 5.9 *	NA
I_Ba_ *k*		− 12.9 ± 2.9	− 12.1 ± 4.7	NA
Inactivation		(n = 18)	(n = 10)	(n = 5)
τ_fast_ (ms)	0 mV	18.7 ± 2.0	24.0 ± 1.8	35.8 ± 4.8^†^
	+ 10 mV	15.0 ± 0.8	25.5 ± 1.6*	38.0 ± 7.2^†^
	+ 20 mV	15.6 ± 0.7	31.0 ± 2.7*	39.9 ± 2.3^†^
A fast/A peak	0 mV	0.51 ± 0.015	0.55 ± 0.033^‡^	0.41 ± 0.034
	+ 10 mV	0.52 ± 0.0092	0.50 ± 0.012^‡^	0.29 ± 0.044^†^
	+ 20 mV	0.46 ± 0.010	0.40 ± 0.013	0.18 ± 0.019^†^
τ_slow_ (ms)	0 mV	165.5 ± 26.6	400.0 ± 102.8	387.7 ± 161.3
	+ 10 mV	123.4 ± 6.6	347.5 ± 75.3*	346.6 ± 135.4
	+ 20 mV	105.0 ± 5.6	251.3 ± 39.5*	244.6 ± 37.8^†^
A slow/A peak	0 mV	0.37 ± 0.010	0.23 ± 0.017*	0.22 ± 0.031^†^
	+ 10 mV	0.40 ± 0.0063	0.31 ± 0.022*	0.30 ± 0.025^†^
	+ 20 mV	0.48 ± 0.010	0.42 ± 0.021	0.34 ± 0.019^†^
A steady/A peak	0 mV	0.15 ± 0.020	0.30 ± 0.039*	0.42 ± 0.013^†^
	+ 10 mV	0.082 ± 0.0081	0.21 ± 0.034*	0.45 ± 0.048^†^
	+ 20 mV	0.055 ± 0.0057	0.60 ± 0.39*	0.48 ± 0.023^†^

Peak current density of activation was measured at + 20 mV for WT and at + 10 mV for R412M. Peak conductance was measured at + 40 mV.

*NA* not available.

*P < 0.05 R412M versus WT.

^†^*P* < 0.05 G406R versus WT.

^‡^*P* < 0.05 R412M versus G406R.

Figure 3B shows plots of current–voltage (IV) relationships of I_Ca,WT_ (black), I_Ca,R412M_ (red), and I_Ca,G406R_ (blue). The voltage at the peak inward currents was more leftward-shifted in I_Ca,R412M_ and I_Ca,G406R_ than in I_Ca,WT_. Figure 3C shows the steady-state activation at various test potentials for I_Ca,WT_, I_Ca,R412M_, and I_Ca,G406R_. Experimental data were fitted with the Boltzmann function described in the “Methods” section (Eq. (2)). The steady-state activation (SSA) curves for I_Ca,R412M_ and I_Ca,G406R_ were significantly shifted toward negative compared to I_Ca,WT_ (Table 1).

#### Ca^2+^ current inactivation decay was significantly delayed by the variants found in TS

The decay of I_Ca_ during depolarization represents fast and slow kinetics, which mainly correspond to Ca-calmodulin-dependent (CDI) and voltage-dependent inactivation (VDI), respectively. Therefore, the time course of Ca^2+^ current decay was fitted to a double exponential function to evaluate time constants for fast and slow components (τ_fast_ and τ_slow_: Eq. (3)). In Fig. 3D, τ_fast_ and τ_slow_ are plotted against test potentials (from 0 to + 20 mV). The τ_fast_ values for I_Ca,R412M_ and I_Ca,G406R_ were significantly larger than those for I_Ca,WT_, while the relative amplitude of the fast component (A_fast_) of I_Ca,R412M_ was comparable to that of I_Ca,WT_ (Table 1). As for the slow component that is largely attributable to VDI, the τ_slow_ values were significantly larger and the relative amplitudes were smaller than I_Ca,WT_ for both in I_Ca,R412M_ and I_Ca,G406R_.

**Figure 4:**
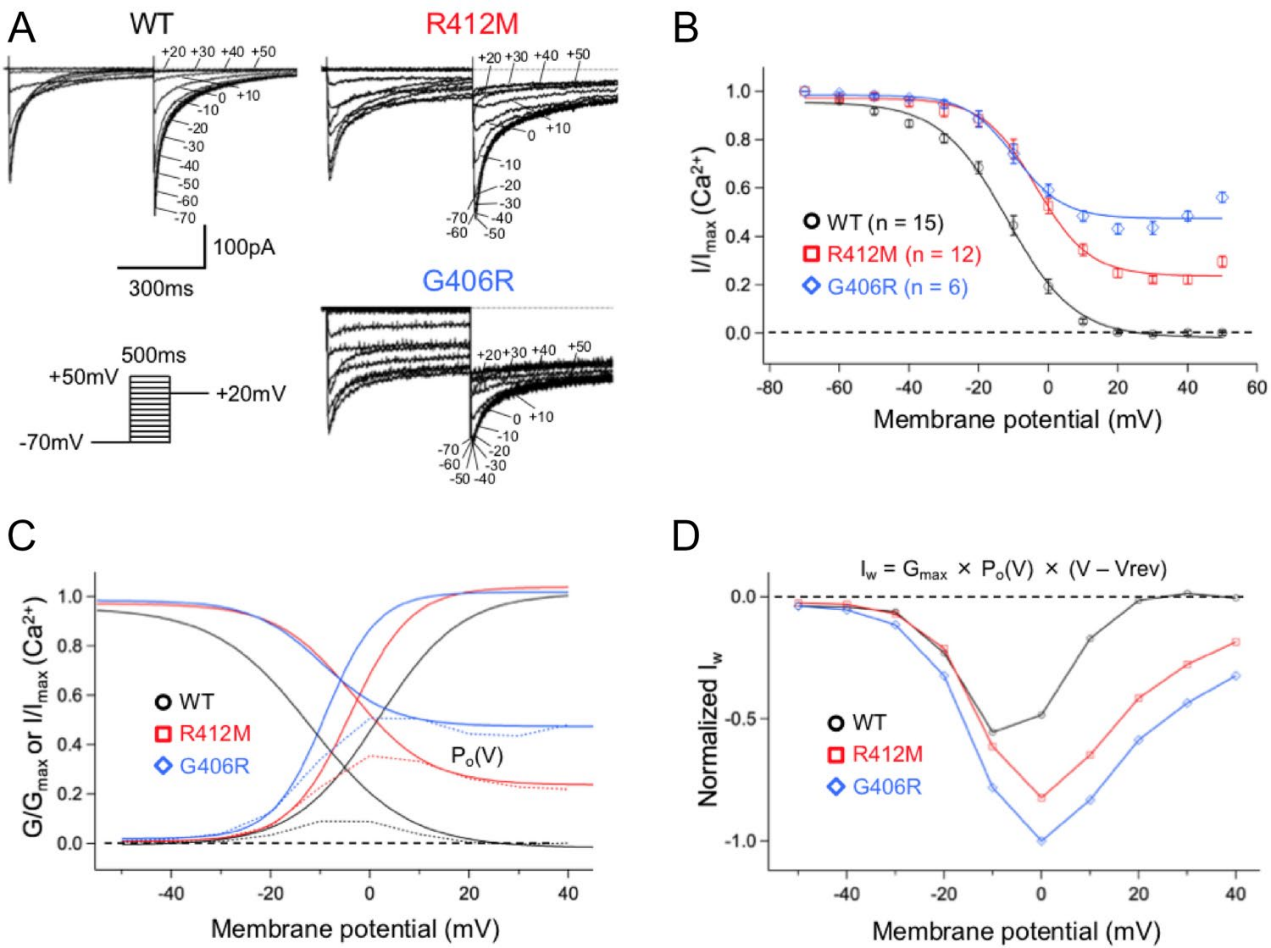
(**A**) Three sets of representative current traces for WT, R412M, and G406R I_Ca_ elicited recorded by a double step pulse protocol in the inset. (**B**) inactivation voltage-dependence curves for WT (black circles, n = 15) and R412M (red squares, n = 12) and G406R (blue diamonds, n = 6) I_Ca_. (**C**) Both activation and inactivation curves are plotted on the same scale. The probability of channel opening P_o_(V) curves were obtained by multiplying G/G_max_ by I/I_max_ (dotted curves). (**D**) The window currents (I_w_) calculated by multiplying P_o_(V) by G_max_ and the driving force (V–V_rev_) and were normalized by the maximum I_w_ at 0 mV in G406R. V_rev_ means reversal potential.

Figure 4A depicts three sets of current traces elicited by a double-pulse voltage protocol (inset panel). Both I_Ca,R412M_ and I_Ca,G406R_ showed the persistent late inward Ca^2+^ currents even at the end of 500-ms test pulse. In Fig. 4B, the peak inward current amplitudes measured at + 20 mV from various test potentials were normalized against their maximal values and are plotted as a function of test voltage. Experimental data were then fitted with the Boltzmann function (Eq. (2) in “Methods” section) to calculate the half-maximal voltage of inactivation (Table 1). While I_Ca,WT_ were completely inactivated at + 20 mV, I_Ca,R412M_ and I_Ca,G406R_ were not inactivated even at + 50 mV (maximal inactivation level of 78% and 54%, respectively). Compared to I_Ca,WT_, the voltage-dependency of steady-state inactivation (SSI) was rightward-shifted to the positive by 7.7 mV in I_Ca,R412M_ and 1.8 mV in I_Ca, G406R_ (Table 1). Figure 4C depicts both activation and inactivation curves for I_Ca,WT_, I_Ca,R412M_, and I_Ca,G406R_ on the same scale. Due to the negative shift of the activation gate and drastic positive shift of the inactivation gate, the window current (I_w_) markedly increased in both R412M (red) and G406R (blue) Ca_v_1.2 channels. Referencing a previous report^6^, we then calculated the I_w_ by first multiplying SSA and SSI to give the open probability of L-type calcium channels (P_o_(V) (Fig. 4C, dotted curves), and then multiplied by G_max_ and the driving force (V–V_rev_) to estimate the amount of I_w_ in WT (black), R412M (red) and G406R (blue) Ca_v_1.2 channels (Fig. 4D: Eq. (4) in “Methods” section). This kind of I_w_ quantification clearly represents that I_w_ of R412M and G406R Ca_v_1.2 channels were larger than that of WT.

#### p.R412M variant mainly affected voltage-dependent inactivation

**Figure 5:**
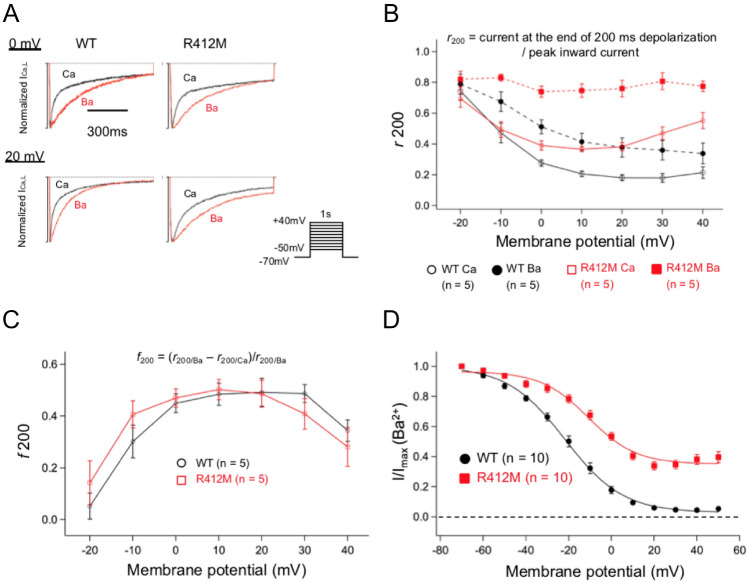
(**A**) Four sets of representative current traces from two different cells expressing either WT (left) or R412M (right) *CACNA1C* as Ca^2+^ (black, I_Ca_) or Ba^2+^ (red, I_Ba_) as a charge carrier at 0 mV (upper panel) and + 20 mV (lower panel). (**B**) Voltage-dependences were evaluated as *r*_200_ (**B**) and *f*_*200*_ (**C**) at various depolarization for WT (n = 5) and R412M (n = 5) and are plotted as a function of test membrane potentials. (**D**) Using the double pulse protocol as shown in Fig. 4A, the voltage-dependency of I_Ba_ inactivation was examined for WT (filled black circles, n = 10) and R412M (filled red squares).

TO further investigate whether p.R412M Ca_v_1.2 channels are affected by alterations of VDI or CDI, we examined characteristics of Ca_v_1.2 using barium (Ba^2+^) as a charge carrier, which allowed us to exclude CDI as previously described^7^. Fig. 5A presents typical current traces of I_Ca_ and I_Ba_ in two different HEK-293 cells expressing WT or R412M Ca_v_1.2 after adjusting the peak inward current levels. We then compared the inactivation time course of I_Ca,WT_ vs. I_Ba,WT_ (left) as well as I_Ca,R412M_ vs. I_Ba,R412M_ (right). When Ca^2+^ ions were present as a charge carrier, the inactivation process was accelerated, indicating the presence of CDI. The degree of CDI was estimated by measuring the ratio of currents remaining at the end of 200-ms depolarization to peak inward currents (*r*_*200*_). The values of *r*_*200*_ thus calculated are plotted as a function of test potential in Fig. 5B. Then, as shown by Eq. (5)**,** the extent of CDI was evaluated as *f*_200_, the fraction of current reduction from I_Ba_ to I_Ca_ (*r*_*200,Ba*_–*r*_*200/Ca*_)^7,8^. The values of *f*_200_ or the component of CDI are plotted against test potentials (Fig. 5C). These values were not significantly different between WT and R412M, indicating that the variant did not significantly affect the CDI but slowed the current decay through the VDI process as shown by time constants of inactivation (Fig. 3D).

Finally, we examined the steady-state inactivation of I_Ba,R412M_ through a double voltage pulse method used in the experiment shown in Fig. 4. Figure 5D shows the voltage-dependency of steady-state inactivation for WT and R412M Ca_v_1.2 currents with Ba^2+^ ion as a charge carrier. The VDI measured as I_Ba,R412M_ was also rightward shifted toward the positive direction by 9.6 mV compared to I_Ba,WT_. Maximal inactivation of I_Ba,R412M_ still remained 66%, indicating that the failure of VDI is indeed the main cause for drastically slowed current decay as previously reported in G406R variant^1,4^.

## Discussion

In the present study, we found a novel *CACNA1C* variant, p.R412M, in a female infant. The variant is located in an α-helical domain between IS6 and AID, close to two previously reported TS variants: p.G406R found in TS1 (exon 8a)^1,2^ or TS2 (exon 8)^2,4^ (Fig. 2C). Functional analyses using a heterologous expression system revealed that p.R412M caused a hyperpolarizing shift of SSA gate (Fig. 3C) and a drastic depolarizing shift of SSI gate (Fig. 4B), resulting in a greater window current (Fig. 4D) and persistent late Ca^2+^ currents at membrane potentials more positive than − 20 mV. Changes in SSA and SSI for I_Ca, G406R_ observed in our experimental condition were consistent with previous reports^1,4,9^. For the measurement of SSI, they used a 2-s pre-pulse duration, while we used a pre-pulse of 500 ms. However, Ferreira et al. indicated that inactivation of I_Ca_ required longer than 5 s to reach steady state^10^. Therefore, only the fast component of inactivation may have been studied in our study. In the study of heterozygous TS2-neo mice, the G406R variant shifted SSI leftward with a pre-pulse of 5 s^6^, which might be more precise for I_Ca, G406R_.

It has been reported that two types of mechanisms, VDI and CDI, are involved in the inactivation of I_Ca_. Experiments using I_Ba_ revealed that p.R412M mainly affected VDI. Therefore, its overall biophysical effects were similar to those of p.G406R, causing the very severe TS-related cardiac phenotypes in our patient.

The IS6-AID linker, where the arginine at position 412 is located in, provides physical interactions between the Ca_V_1.2 β-subunit and the channel pore. This interaction between two domains is thought to be pivotal for the smooth VDI gating of the channel^11^. Previous studies showed that an increased rigidity of the IS6-AID linker decelerates the time course of Ca_v_1.2 VDI^11,12^. This rigid stabilization was proposed to be the pathophysiological mechanism behind the G406R variant (both in TS1 and TS2), which critically slowed the inactivation kinetics^12^. As the topological location is close to these classical TS mutations, p.R412M may also increase the rigidity of the IS6-AID linker, thereby slowing the VDI process.

Our index patient presented a marked QT-prolongation that was longer than the QT-intervals in TS1 patients^1^, with a more severe cardiac phenotype than those observed in reported TS1 cases. Therefore, the clinical features of our patient resembled those of TS2 patients. The variant p.G406R in TS1 is translated from exon 8a, and the variant p.R412M found in our patient is translated from exon 9. It has been reported that expression levels of the *CACNA1C* transcript containing an alternatively spliced exon 8a represent approximately only 20% of the total cardiac *CACNA1C* transcript, while exon 9 is 100% translated since no alternative transcripts exist^1,2,4,13^. In contrast, the majority of cardiac *CACNA1C* transcripts contain the mutually spliced exon 8. Thus, TS2 patients bearing p.G406R in exon 8 might show severe cardiac phenotypes comparable to p.R412M^2,4^. These differences in genetic backgrounds may account for the various severity of clinical outcomes^1,2,4^.

Although we observed that the R412M mutation did not affect the CDI largely just as the p.G406R variant previously found in classical TS patients, Dick et al. demonstrated that the G406R mutation caused significant defects in CDI of the channel^8^. They co-expressed β2a auxiliary subunit which decreased VDI in Ca^2+^ channels in order to examine CDI precisely. However, we used β2b auxiliary subunit according to the previously reported “Methods” section^7^. Therefore, we could not deny the CDI impairment by R412M in in situ hearts.

TS is an extremely rare syndromic disease, and approximately 50 cases have been described to date^2^. Later on, variants located outside of exon 8/8a were identified: p.E407G, p.E407A, p.G419R, p.S643F, p.C1021R, p.E1115K, p.I1166T, p.A1473G, and p.G1911R in atypical TS patients (Fig. 2C)^14–22^. More recently, a wide variety of phenotypes in *CACNA1C* variant carrier have been reported, including those expressing only cardiac features or even long QT syndrome (LQT8) alone^17,23–25^.

Considering that the heart is the most frequently affected organ in TS, ECG would be a useful tool to diagnose and determine the prognosis. As seen in our patient, key features are: extremely prolonged QT intervals (QTc > 600 ms); 2:1 atrio-ventricular block and recurrent TdP; and macroscopic T-wave alternans. These ECG changes in TS are more prominent compared to the other congenital LQTS’s. When encountering such cases, irrespective of presence or absence of extra-cardiac phenotypes, it would be of clinical importance to conduct genetic testing including that of *CACNA1C*.

## Limitation

We could not completely exclude the possibility that other variants might contribute to extracardiac symptoms because a whole exome or genome sequencing was not done. We conducted an electrophysiological study using *CACNA1C* cDNA containing alternative exon 8a. We did not confirm whether a *CACNA1C* variant, p.R412M, caused the same electrophysiological effects on Ca_v_1.2 when exon 8 expressed. In addition, for the measurement of SSI, we employed a short pre-pulse duration, which may have caused ‘quasi’-steady state inactivation and window current.

## Conclusion

In a female infant with the atypical TS mimicking TS2 features and sudden cardiac death, we identified a novel heterozygous *CACNA1C* variant, p.R412M. Functional assay of p.R412M showed a significant VDI deceleration of Ca_V_1.2 channel, consistent with the TS1 variant p.G406R. Our study indicates that patients with TS who carry pathogenic *CACNA1C* variants should be carefully observed to prevent unexpected sudden cardiac death.

## Methods

### Genetic screening

In accordance with study protocol that was approved by the review board at Shiga University of Medical Science and complied the principles of the Declaration of Helsinki, genetic analysis was performed after written informed consent was obtained from the parent of the proband. Genomic DNA was extracted from peripheral blood leukocytes. Coding and splice-site regions of 56 genes including LQTS-related genes (Supplementary Table 1) were all screened via targeted gene sequencing using a next generation sequencer (Miseq, Illumina, San Diego, CA, USA)^26^. For confirmation, direct DNA sequencing was conducted on an ABI PRISM-3130 Sequencer (Applied Biosystems, Foster City, CA, USA). The GenBank accession number of *CACNA1C* is NM_000719.6. We confirmed the pathogenicity of the variants according to Varsome (https://varsome.com/) and ACMG guideline for the interpretation of sequence variants^5^.

### Mutagenesis and transient transfection

The human wild-type (WT) *CACNA1C* cDNA (NM_000719), which contains alternative exon 8a, in a pcDNA vector, and cDNAs of *CACNB2b* and *CACNA2D1*, both cloned into a pcDNA3.1 vector, were used. The vectors were kindly donated by Prof. Charles Antzelevitch (Lankenau Institute for Medical Research, USA). Site-directed mutagenesis was performed using a QuickChange II XL kit (Stratagene, La Jolla, CA, USA). Mutated genes, *CACNA1C* R412M and G406R, were functionally expressed in human embryonic kidney (HEK) 293 cells. HEK-293 cells were co-transfected with WT or mutant *CACNA1C* cDNAs (1 µg each) along with *CACNB2b* (1 µg), *CACNA2D1* (1 µg), and Green Fluorescence Protein (GFP, 0.25 µg) using 6 µl of Fugene 6 (Roche Diagnostics, Indianapolis, IN, USA). Cells were employed for electrophysiological experiments 24–36 h after transfection.

### Electrophysiology

A whole-cell mode patch-clamp technique was employed to measure WT and mutant Ca^2+^ currents at 25–26 °C using an Axopatch 200B patch-clamp amplifier (Axon Instruments, Foster City, CA, USA). We used 1 µM of nisoldipine to dissect reconstituted L-type calcium currents (I_Ca_) by digital subtraction. The extracellular (bath) solution contained (mmol/L): 130 NMDG-Cl, 5 KCl, 15 CaCl_2_ (or BaCl_2_), 1 MgCl_2_, and 10 HEPES. The pipette solution contained (mmol/L): 120 CsCl, 2 MgCl_2_, 2 MgATP, 10 HEPES, 5 CaCl_2_, and 10 EGTA (pH was adjusted to 7.25 with CsOH)^7^. Free Ca^2+^ concentration in the pipette solution was adjusted to be 100 nM by adding appropriate amounts of CaCl_2_ calculated by the Patcher’s Power Tools package (Igor Pro^TM^Tool software, version 1.0.6, 1997, © 1995–1997, Dr Francisco Mendez, Dept. of Membrane Biophysics, MPIbpc Gottingen, Germany). Patch pipettes were prepared using a P-97 puller (Sutter Instruments, Novato, CA, USA) and were fire-polished to a final resistance of 2–3 MΩ.

To study the voltage dependence of channel activation, a single-step voltage protocol was used: depolarizing test pulses with 1-s durations were applied between − 50 and + 40 mV in 10 mV increments from a holding potential of − 70 mV. Data were analyzed using Clampfit (Axon Instruments, Sunnyvale, CA, USA) and fitted with Igor Pro or Prism 7 (GraphPad Software Inc. ver.9). Peak current densities at each potential were obtained by dividing the cell capacitance.

Ca current–voltage (I_Ca_–V) curves were fitted with the Boltzmann function of the following form:

1 $${\text{I}}_{{{\text{Ca}}}} \left( {\text{V}} \right) \, = {\text{ G}}_{{{\text{max}}}} \times \, \left( {{\text{V }}{-}{\text{ V}}_{{{\text{rev}}}} } \right)/\left\{ {{1 } + {\text{ exp }}\left[ {\left( {{\text{V}}_{h} {-}{\text{ V}}} \right)/k} \right]} \right\},$$

where I_Ca_(V) indicates I_Ca_ (pA/pF) at the membrane potential of V (mV), G_max_ is maximum conductance density, V_rev_ is the reversal potential, V_*h*_ is the activation midpoint voltage, and *k* is the slope factor which determines the voltage dependence of channel activation.

The steady-state activation (SSA) and inactivation (SSI) curves were fitted with Boltzmann function of the following form:

2 $$\begin{aligned} {\text{G}}/{\text{G}}_{{{\text{max}}}} \left( {\text{V}} \right) & = {1} - { 1}/\left\{ {{1 } + {\text{ exp }}\left[ {\left( {{\text{V}}{-}{\text{V}}_{h} } \right)/k} \right]} \right\}\quad {\text{or}} \\ {\text{I}}/{\text{I}}_{{{\text{max}}}} \left( {\text{V}} \right) \, & = { 1}/\left\{ {{1 } + {\text{ exp }}\left[ {\left( {{\text{V}}_{{}} {-}{\text{ V}}_{h} } \right)/k} \right]} \right\}, \\ \end{aligned}$$

where G/G_max_(V) indicates normalized I_Ca_ SSA, and I/I_max_(V) SSI at the membrane potential V, respectively.

To obtain the inactivation time constant, the time course of inactivating currents for the first 300 ms at 0, + 10, and + 20 mV were fitted with a double exponential function:

3 $${\text{I}}_{{{\text{Ca}}}} \left( t \right) \, = {\text{A}}_{0} + {\text{A}}_{{\text{f}}} \left[ {{1 }{-}{\text{ exp }}( - t/\tau_{{\text{f}}} )} \right] + {\text{A}}_{{\text{s}}} \left[ {{1 }{-}{\text{ exp }}( - t/\tau_{{\text{s}}} )} \right],$$

where I_Ca_(*t*) is the calcium current at time *t* (ms), A is the current amplitude, and τ (ms) is the inactivation decay time constant.

The probability of a channel opening at the membrane potential V (P_o_(V)) was obtained by multiplying G/G_max_(V) by I/I_max_(V). The window currents (I_W_) were quantified by the equation.

4 $${\text{I}}_{{\text{w}}} = {\text{G}}_{{{\text{max}}}} \times {\text{ P}}_{{\text{o}}} \left( {\text{V}} \right) \, \times \, \left( {{\text{V }} - {\text{ V}}_{{{\text{rev}}}} } \right)$$

To analyze the underlying mechanisms of the inactivation of Ca^2+^ currents, WT or R412M Ca_V_1.2 currents were measured using extracellular (bath) CaCl_2_ or BaCl_2_ solutions in the same cells. Then, fractions of peak currents remaining after 200-ms depolarization were normalized to a peak current (*r*_200_) at various test potentials. The fractions of CDI were calculated as^7,8^:

5 $$f_{{{2}00}} = \, \left( {r_{{{2}00/{\text{Ba}}}} {-}r_{{{2}00/{\text{Ca}}}} } \right)/r_{{{2}00/{\text{Ba}}}} .$$

### Statistical analysis

All analyses were performed using the SPSS 22.0 statistical package (IBM, Corp., Armonk, NY, USA). Differences between the two groups were evaluated using Mann–Whitney’s *U* test. Differences were accepted as statistically significant for *p* values < 0.05. For comparisons among the three groups, one-way ANOVA or Kruskal–Wallis tests were performed. Bonferroni correction was used for post hoc pairwise comparison. Continuous patch-clamp data are expressed as a mean (± SEM or 95% CI).

## Supplementary Information

Supplementary Information.

## Data Availability

The datasets of the novel variant p.R412M in *CACNA1C* are registered in the dbSNP repository (ss2137544377, https://www.ncbi.nlm.nih.gov/SNP/snp_ss.cgi?ss=2137544377). Other datasets generated and analyzed during the current study are available from the corresponding author on reasonable request.
