# Supplementary material for: Increased CaV1.2 late current by a CACNA1C p.R412M variant causes an atypical Timothy syndrome without syndactyly
Source: Sci Rep. 2022 Nov 8;12:18984. doi: 10.1038/s41598-022-23512-2 (PMC9643354; doi:10.1038/s41598-022-23512-2)
Supplement: Supplementary file 1 — Supplementary Information. [file 41598_2022_23512_MOESM1_ESM.docx]

**Supplementary Table 1:** Targeted genes for next generation sequencer

| ***AKAP9*** | *DSC2* | *KCNE5* | *RANGRF* |
| --- | --- | --- | --- |
| ***ANKB*** | *DSG2* | ***KCNH2*** | *RYR2* |
| ***CACNA1C*** | *DSP* | *KCNIP2* | *SCN10A* |
| *CACNA2D1* | *GJA1* | ***KCNJ2*** | *SCN1B* |
| *CACNB2* | *GJA5* | *KCNJ3* | *SCN2B* |
| ***CALM1*** | *GPD1L* | ***KCNJ5*** | *SCN3B* |
| ***CALM2*** | *HCN4* | *KCNJ8* | ***SCN4B*** |
| ***CALM3*** | *JUP* | *KCNN2* | ***SCN5A*** |
| *CAMK2D* | *KCNA5* | ***KCNQ1*** | *SLC8A1* |
| *CASQ2* | *KCND3* | *LMNA* | ***SNTA1*** |
| ***CAV3*** | ***KCNE1*** | *MYBPC3* | *TCAP* |
| *CHRM2* | ***KCNE2*** | *MYH6* | *TMEM43* |
| *CTNNA3* | *KCNE3* | *NCS1* | *TRDN* |
| *DPP6* | *KCNE4* | *PKP2* | *TRPM4* |

* Gene names written in **BOLD** are established causative gene of long QT syndrome.
